# Quantitative Analysis for the Delineation of the Subthalamic Nuclei on Three-Dimensional Stereotactic MRI Before Deep Brain Stimulation Surgery for Medication-Refractory Parkinson’s Disease

**DOI:** 10.3389/fnhum.2022.829198

**Published:** 2022-02-22

**Authors:** Chun-Yu Su, Alex Mun-Ching Wong, Chih-Chen Chang, Po-Hsun Tu, Chiung Chu Chen, Chih-Hua Yeh

**Affiliations:** ^1^Department of Medical Imaging and Intervention, Chang Gung Memorial Hospital, Linkou, Taiwan; ^2^College of Medicine, Chang Gung University, Taoyuan, Taiwan; ^3^Department of Diagnostic Radiology, Chang Gung Memorial Hospital, Keelung, Taiwan; ^4^Department of Neurosurgery, Chang Gung Memorial Hospital, Linkou, Taiwan; ^5^Department of Neurology, Chang Gung Memorial Hospital, Linkou, Taiwan; ^6^Neuroscience Research Center, Chang Gung Memorial Hospital, Linkou, Taiwan

**Keywords:** subthalamic nuclei, Parkinson’s disease, deep brain stimulation, signal-to-noise ratio, contrast, signal difference-to-contrast ratio

## Abstract

Delineation of the subthalamic nuclei (STN) on MRI is critical for deep brain stimulation (DBS) surgery in patients with Parkinson’s disease (PD). We propose this retrospective cohort study for quantitative analysis of MR signal-to-noise ratio (SNR), contrast, and signal difference-to-noise ratio (SDNR) of the STN on pre-operative three-dimensional (3D) stereotactic MRI in patients with medication-refractory PD. Forty-five consecutive patients with medication-refractory PD who underwent STN-DBS surgery in our hospital from January 2018 to June 2021 were included in this study. All patients had whole-brain 3D MRI, including T2-weighted imaging (T2WI), T2-weighted fluid-attenuated inversion recovery (FLAIR), and susceptibility-weighted imaging (SWI), at 3.0 T scanner for stereotactic navigation. The signal intensities of the STN, corona radiata, and background noise were obtained after placing regions of interest (ROIs) on corresponding structures. Quantitative comparisons of SNR, contrast, and SDNR of the STN between MR pulse sequences, including the T2WI, FLAIR, and SWI. Subgroup analysis regarding patients’ sex, age, and duration of treatment. We used one-way repeated measures analysis of variance for quantitative comparisons of SNR, contrast, and SDNR of the STN between different MR pulse sequences, and we also used the dependent *t*-test for the *post hoc* tests. In addition, we used Mann–Whitney U test for subgroup analyses. Both the contrast (0.33 ± 0.07) and SDNR (98.65 ± 51.37) were highest on FLAIR (all *p* < 0.001). The SNR was highest on SWI (276.16 ± 115.5), and both the SNR (94.23 ± 31.63) and SDNR (32.14 ± 17.23) were lowest on T2WI. Subgroup analyses demonstrated significantly lower SDNR on SWI for patients receiving medication treatment for ≥13 years (*p* = 0.003). In conclusion, on 3D stereotactic MRI of medication-refractory PD patients, the contrast and SDNR for the STN are highest on FLAIR, suggesting the optimal delineation of STN on FLAIR.

## Introduction

Most patients with Parkinson’s disease (PD) are treated with medication, and a multitude of dopamine-enhancing agents is available as the therapeutic option (Armstrong and Okun, 2020). However, deep brain stimulation (DBS) has been successfully used to treat PD among patients who do not adequately respond to pharmacologic treatment, or who have intolerable medication-induced complications, which may be more severe than the motor impairment of the disease itself (Perestelo-Perez et al., 2014). Subthalamic nuclei (STN) are the most used targets of electrode implantation in patients with PD (Vizcarra et al., 2019). Because the STN are indiscernible on CT images and conventional MR images at 1.5 T, STN targeting has conventionally been performed indirectly by predicting the location of the STN according to coordinates derived from atlases (Tu et al., 2018). The drawback of the indirect targeting method, however, is that STN sizes, shapes, and positions vary between patients (Chandran et al., 2016).

With the advancement of MRI imaging techniques, delineation of the deep brain nuclei became possible on MRI at 3.0 T. Currently, the direct targeting method, which involves attempting to locate the STN in each patient, has become the mainstream targeting technique for DBS surgery (Larson et al., 2012). Compared with the adjacent white matter structures, the STN is relatively hypointense on T2-weighted imaging (T2WI), T2-weighted fluid-attenuated inversion recovery (FLAIR), and susceptibility-weighted imaging (SWI). STN is typically 3 mm lateral to the lateral border of the red nucleus, and 2 mm inferior to the superior border of the red nuclei (Andrade-Souza et al., 2008); however, the STN remain difficult to image because of their biconvex shape, small size, and its oblique spatial orientation (Ashkan et al., 2007). In our hospital, whole-brain three-dimensional (3D) stereotactic MR with T1-weighted imaging (T1WI), T2WI, FLAIR, and SWI are obtained for trajectory planning before DBS surgery (Chandran et al., 2016). Delineating the STN on MRI is vital for the direct targeting method employed in the DBS surgery. The signal intensities (SIs) of the STN and surrounding white matter structures, however, vary on by MR pulse sequences, which may influence the ability to differentiate between these structures (Wolff and Balaban, 1997).

In the field of diagnostic imaging, the quality of images and the ability to demonstrate the target lesion are crucial. The MR imaging quality depends on both the signal intensity (SI) of the human body structures and the noise caused by the thermally driven Brownian motion of electrons within the body’s conducting tissue and within the receiving coil itself (Kaufman et al., 1989). Signal-to-noise ratio (SNR) is one of the standardized parameters for quantitative measurement and comparison of image quality. Contrast is the ratio of the difference in SI between two regions, which can reflect the human eyes’ ability to differentiate between these two regions (Wolff and Balaban, 1997). Furthermore, signal difference-to-noise ratio (SDNR) is calculated by dividing the difference in SI by noise and is a display-independent parameter that reflects the contrast-generating ability of a pulse sequence (Wolff and Balaban, 1997; Pijl et al., 2004). Contrast and SDNR are both commonly used to measure the ability to delineate a structure on MR pulse sequences.

In this retrospective cohort study, we compared the delineation of the STN on multiple MR pulse sequences. Additionally, studies of STN delineation on MRI have had limited sample sizes or were based on MRI of healthy participants (Ashkan et al., 2007; O’Gorman et al., 2011). Therefore, the purpose of this study was to propose a quantitative analysis of SNR, contrast, and SDNR for STN on 3D stereotactic MRI before DBS surgery in patients with medication-refractory PD.

## Materials and Methods

### Patients

This study was approved by our institution’s institutional review board (IRB NO: 202101300B0). We retrospectively included 45 consecutive patients with medication-refractory PD who underwent STN DBS surgery in our hospital from January 2018 to June 2021. The following clinical data were collected through medical chart review: basic demographics, duration of medication treatment, history of other chronic diseases, and report of dopamine scan.

### MRI Technique and Deep Brain Stimulation Trajectory Planning

MRI was performed on a 3.0 T MR system (Ingenia, Philips Medical Systems, Best, Netherlands) with the patient in the supine position. Whole-brain 3D turbo spin-echo T1WI, T2WI, and FLAIR were performed using a 15-channel head coil (dStream HeadSpine coil, Philips Medical Systems, Best, Netherlands). A total of 160 slices of axial sections without intersection gap in the orientation parallel to the AC–PC line orientation were obtained. Axial SWI with the same coverage was also performed. The detailed parameters of the MR pulse sequences are listed in Table 1. Immediately before the STN-DBS surgery, a whole-brain stereotactic non-enhanced CT in 1-mm slice thickness was also performed after application of the Cosman-Roberts-Wells frame (Integra Radionics, Burlington, MA, United States). Images of the 3D MRI and stereotactic CT were both transferred to a stereotactic workstation (BrainLab AG, Munich, Germany) for imaging fusion and target planning.

**TABLE 1:** Detailed parameters of MR pulse sequence for three-dimensional stereotactic MRI for preoperative evaluation of deep brain stimulation surgery.

	T1WI	T2WI	FLAIR	SWI
TR (msec)	6.1	2000	4800	30
TE (msec)	2.8	136	268	7.2
Flip angle (deg)	8	90	40	17
Slices	160	160	160	160
Thickness (mm)	1	1	1	2
Gap (mm)	0	0	0	-1
Bandwidth (Hz)	334	890	890	254
Field of view (mm)	240 × 200	240 × 200	240 × 200	230 × 179
Matrix size	240 × 200	240 × 200	240 × 200	328 × 257
Scan time (min:sec)	04:06	05:26	07:41	05:07

*T2WI, T2-weighted imaging; T1WI, T1-weighted imaging; FLAIR, fluid-attenuated inversion recovery; SWI, susceptibility-weighted imaging.*

### Data Postprocessing

**FIGURE 1:**
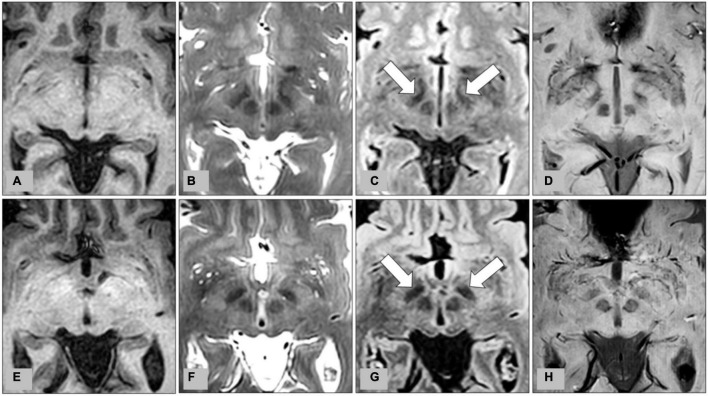
Representative images from preoperative MRI of two patients with medication-refractory Parkinson’s disease at the level of the subthalamic nucleus (STN). **(A)** T1-weighted imaging (T1WI), **(B)** T2-weighted imaging (T2WI), **(C)** fluid-attenuated inversion recovery (FLAIR), and **(D)** susceptibility-weighted imaging (SWI) from MRI of a 56-year-old male patient, and corresponding **(E)** T1WI, **(F)** T2WI, **(G)** FLAIR, and **(H)** SWI from MRI of a 62-year-old male patient. Bilateral STN are indicated by arrows on FLAIR **(C,G)**.

**FIGURE 2:**
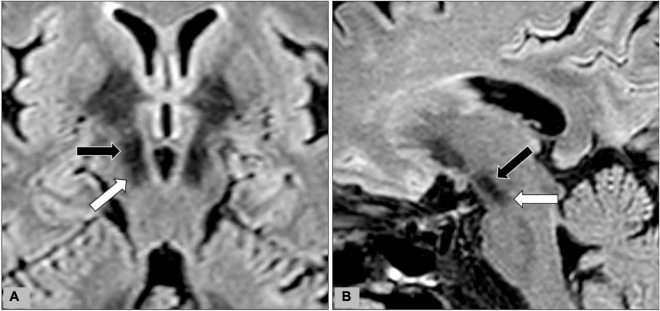
During data-postprocessing, we selected the level of axial image with the optimal visualization of subthalamic nuclei (black arrows) after a review of the T2WI, FLAIR, and SWI images. Oblique coronal **(A)** and sagittal **(B)** reformation images were rechecked to ensure that the substantia nigra (white arrow) was not covered on the selected image.

**FIGURE 3:**
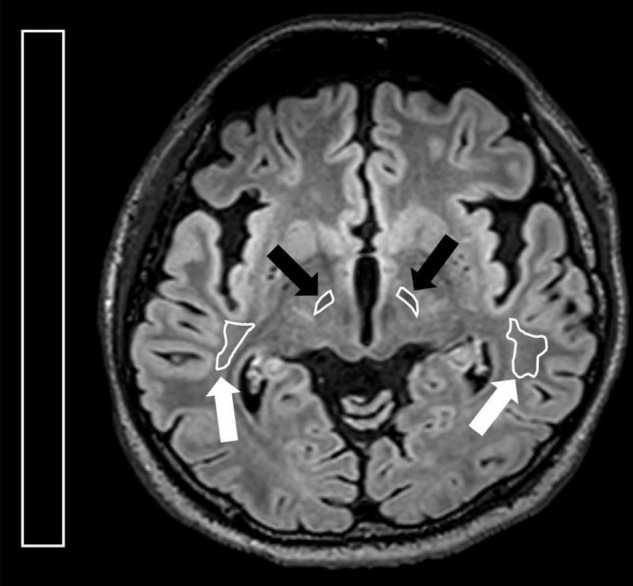
Illustration for regions of interest (ROIs) placed on the axial FLAIR image. On the axial image with the optimal visualization of both subthalamic nucleus, ROIs were placed at bilateral STN (black arrows) and corona radiata (white arrows). A rectangular ROI of the background area with an area greater than 10.0 cm^2^ was placed on the right side of the image.

All MR images were analyzed on a postprocessing workstation (IntelliSpace Portal, Philips Medical Systems, Best, Netherlands). First, the level of axial MR image with the optimal visualization of both STN and red nuclei was selected after a review of the T2WI, FLAIR, and SWI (Figure 1). Oblique coronal and sagittal reformation images were rechecked to ensure that the substantia nigra was not covered on the selected image (Figure 2). On the selected images, regions of interest (ROIs) were placed on bilateral STN and adjacent corona radiata. A rectangular ROI of the background area with a long axis perpendicular to the phase-encoding direction and with an area greater than 10.0 cm^2^ was placed on the right aspect of the images. Two radiologists with experience in neuroimaging for more than 10 years independently reviewed the images and determined ROIs for all patients. Figure 3 illustrates a representative example of the ROI placed on the FLAIR image.

In the next step, we calculated the SNR, contrast, and SDNR using the following formulas (Wolff and Balaban, 1997; Pijl et al., 2004).


$$SNR=\frac{meansignalintensity\left(SI\right)ofSTN\;}{standarddeviation\left(SD\right)ofbackgroundnoise}$$

$$Contrast=\frac{meanSIofcoronaradiata-meanSIofSTN}{meanSIofcoronaradiata\;}$$

$$SDNR=\frac{meanSIofcoronaradiata-meanSIofSTN}{SDofbackgroundnoise}$$

### Evaluation for Subthalamic Nuclei Border Delineation

**FIGURE 4:**
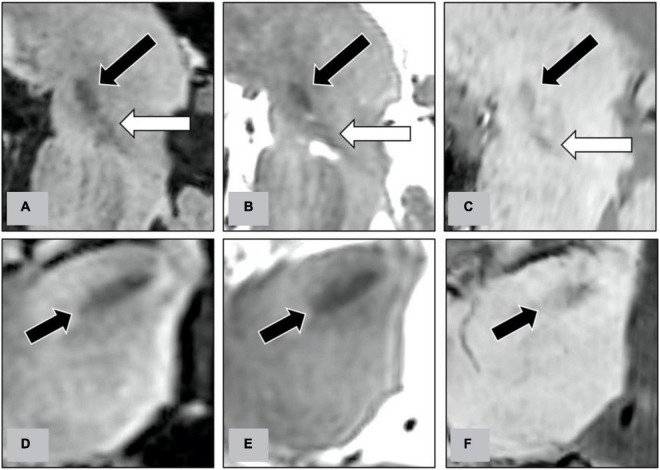
Along the trajectory of STN-DBS electrodes, we reconstructed the oblique sagittal images on FLAIR **(A)**, T2WI **(B)**, and SWI **(C)**. On the oblique sagittal images, we evaluate the delineation between STN (indicated by black arrows on figures) and substantia nigra (indicated by white arrows). We also reconstructed the oblique axial images perpendicularly to the trajectory at the level of the STN on FLAIR **(D)**, T2WI **(E)**, and SWI **(F)**. On the oblique axial images, we evaluate the delineation of the lateral border of STN (indicated by black arrows).

Along the trajectory of STN-DBS electrodes, we reconstructed the oblique sagittal images on different MR pulse sequences. We also reconstructed the oblique axial images perpendicularly to the trajectory at the level of the STN (Figure 4). On the oblique sagittal images, we evaluate the delineation between STN and substantia nigra. On the oblique axial images, we evaluate the delineation of the lateral border of STN. A fixed-points scale was used for qualitative evaluation of the STN delineation (Score 1: delineation not visible. Score 2: delineation barely visible with highly blurred margin. Score 3: visible delineation with moderately blurred margin. Score 4: delineation with slightly blurred margin. Score 5: delineation with excellent shapely defined margin.). The two neuroradiologists evaluated these images independently. And discrepancy in scoring was solved by consensus.

### Statistical Analyses

Intraclass correlation coefficient (ICC) was calculated to represent the interobserver agreement. One-way repeated measures analysis of variance (ANOVA; Mishra et al., 2019) was used to compare the SNR, contrast, and SDNR for STN between multiple MR pulse sequences. Dependent *t*-tests were used for the *post hoc* tests of one-way repeated measures ANOVA. Subgroup analyses were performed according to the sex, mean age (<65 years vs. ≥65 years), and mean duration of medication treatment (<13 years vs. ≥13 years) of these 45 patients. The Mann–Whitney U test was used for non-parametric comparisons in subgroup analyses. A *p-value* of <0.05 indicated statistically significant differences, and the *p*-values were adjusted using the Bonferroni multiple testing correction for multiple comparisons in the *post hoc* tests and subgroup analyses. We also calculated the Cramer’s V coefficient to represent the correlations of STN delineation between different MR pulse sequences. All statistical analyses were performed using RStudio software (version 1.4.0, Boston, MA, United States) (Hackenberger, 2020).

## Results

### Patients

This retrospective cohort included 45 patients with medication-refractory PD. Fourteen patients were women, and 31 patients were men. The mean age of these patients was 62.09 ± 9.17 (mean ± SD, range = 38–73) years. The mean duration of medication treatment for PD was 13.09 ± 4.69 (range = 6–25) years. Five patients had diabetes, and 15 patients had hypertension. None of the patients had other major systemic disorders. From the dopamine scan, 19 patients were determined to have a right-side predominant disease, 11 patients had the left-side predominant disease, and the other 15 patients had the bilateral symmetric disease.

### Signal Intensity and Interobserver Agreement

The mean SIs of STN and corona radiata were normally distributed on Q–Q plots. The ICC between the SIs of the STN measured by two observers was 0.796 on T2WI, 0.899 on FLAIR, and 0.877 on SWI. The ICC for corona radiata was 0.751 on T2WI, 0.856 on FLAIR, and 0.854 on SWI. These results suggested satisfactory interobserver agreement. The SI of the STN was 314.78 ± 49.33 on T2WI, 659.25 ± 210.71 on FLAIR, and 720.97 ± 122.43 on SWI (Table 2). The SI of the corona radiata was highest on FLAIR (986.29 ± 301.77), followed by the SI on SWI (886.02 ± 141.8) and SI on T2WI (420.62 ± 54.38). Background noise was relatively low on all three pulse sequences. Mean noise was largest on T2WI (2.41 ± 0.53) and smallest on FLAIR (0.86 ± 0.53), but the variation of the background noise was largest on SWI (1.28 ± 1.22).

**TABLE 2:** Signal intensity measurements for regions of interest (ROI) locations in 45 patients with medication-refractory Parkinson’s disease.

	Signal intensity		
	T2WI	FLAIR	SWI
**Target structures**			
STN	314.78 ± 49.33	659.25 ± 210.71	720.97 ± 122.43
Surround structure			
Corona radiata	420.62 ± 54.38	986.29 ± 301.77	886.02 ± 141.8
Background area			
Background noise	2.41 ± 0.53	0.86 ± 0.53	1.28 ± 1.22

T2WI, T2-weighted imaging; FLAIR, fluid-attenuated inversion recovery; SWI, susceptibility-weighted imaging; STN, Subthalamic nucleus. Data are presented as mean ± SD.

### Signal-To-Noise Ratio

**FIGURE 5:**
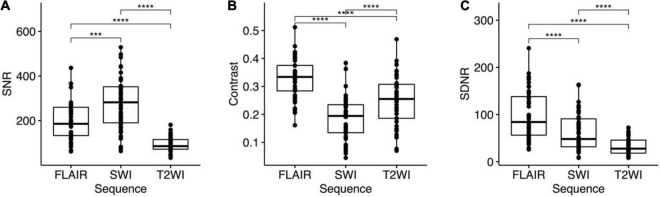
One-way repeated measures analysis of variance (ANOVA) and *post hoc* tests for **(A)** signal-to-noise ratio (SNR), **(B)** contrast, and **(C)** signal difference-to-noise ratio (SDNR) the STN on FLAIR, SWI, and T2WI. Bonferroni multiple testing correction for multiple comparisons in the *post hoc* tests. All the *p* < 0.001 for ANOVA and *post hoc* tests of SNR, contrasts, and SDNR. ***p ≤ 0.001 and ****p ≤ 0.0001.

The SNR of STN was also highest on SWI (276.16 ± 115.5), followed by that on FLAIR (196.18 ± 86.45). Because the lowest STN SI was observed on T2WI, the SNR was also lowest on T2WI (94.23 ± 31.63). Repeated measures ANOVA (Figure 5A) revealed significant differences between the SNR of these three pulse sequences (*p* < 0.001). *Post hoc* tests using a dependent *t*-test also demonstrated significant differences between T2WI and FLAIR, between FLAIR and SWI, and between T2WI and SWI (all *p* < 0.001).

### Contrast and Signal Difference-To-Noise Ratio

Both the contrast and the SDNR of the STN were highest on FLAIR (contrast: 0.33 ± 0.07; SDNR: 98.65 ± 51.37). The contrast of the STN was smallest on SWI (0.18 ± 0.08), but the SDNR was smallest on T2WI (32.14 ± 17.23; Table 3). Significant differences of contrast were noted between the three pulse sequences on repeated measures ANOVA (*p* < 0.001) and *post hoc* tests (all *p* < 0.001; Figure 5B). For the SDNR (Figure 5C), significant differences were also noted on repeated measures ANOVA and *post hoc* tests (all *p* < 0.001).

**TABLE 3:** SNR, contrast, and SDNR for subthalamic nucleus in 45 patients with medication-refractory Parkinson’s disease.

	T2WI	FLAIR	SWI	*p*
SNR	94.23 ± 31.63	196.18 ± 86.45	276.16 ± 115.5	<*0.001*
Contrast	0.25 ± 0.09	0.33 ± 0.07	0.18 ± 0.08	<*0.001*
SDNR	32.14 ± 17.23	98.65 ± 51.37	62.68 ± 38.55	<*0.001*

T2WI, T2-weighted imaging; FLAIR, fluid-attenuated inversion recovery; SWI, susceptibility-weighted imaging; SNR, signal-to-noise ratio; SDNR, signal difference-to-contrast ratio. Data are presented as mean ± SD.

### Subgroup Analyses

No significant difference in SNR, contrast or SDNR of the STN on the three pulse sequences were noted between patients of different sex (14 women and 31 men) or age [<65 years (*n* = 23) and ≥65 years (*n* = 22); Table 4]. However, after Bonferroni correction, the SDNR on SWI was significantly lower among the 25 patients who had been treated with medication for ≥13 years (mean = 46.83) than it was among the 20 patients who had been treated with medication for <13 years (mean = 82.50, *p* = 0.003). The SDNR on FLAIR (*p* = 0.032) and T2WI (*p* = 0.207) also trended lower in patients with a longer history of medication treatment, but the difference was non-significant.

**TABLE 4:** Subgroup comparison of SNR, contrast, and SDNR for STN in 45 patients with medication-refractory Parkinson’s disease.

	Sex			Age (years)			Medication treatment (years)		
	Woman (*n* = 14)	Man (*n* = 31)	*p*	<65 (*n* = 23)	≧65 (*n* = 22)	*p*	<13 (*n* = 20)	≧13 (*n* = 25)	*p*
**SNR**									
T2WI	95.52	93.65	*0.952*	88.16	100.57	*0.194*	103.38	86.91	*0.199*
FLAIR	227.96	181.83	*0.362*	172.08	221.38	*0.115*	200.32	192.87	*0.883*
SWI	253.63	286.34	*0.313*	273.06	279.42	*0.813*	317.75	242.90	*0.042*
**Contrast**									
T2WI	0.27	0.24	*0.375*	0.26	0.24	*0.464*	0.27	0.23	*0.191*
FLAIR	0.32	0.33	*0.913*	0.34	0.32	*0.350*	0.35	0.31	*0.124*
SWI	0.19	0.18	*0.637*	0.19	0.18	*0.218*	0.20	0.17	*0.136*
**SDNR**									
T2WI	36.24	30.30	*0.325*	31.77	32.54	*0.937*	38.13	27.35	*0.032*
FLAIR	108.10	94.38	*0.672*	91.16	106.48	*0.437*	111.15	88.65	*0.207*
SWI	65.37	61.47	*0.781*	66.03	59.18	*0.361*	82.50	46.83	0.003*

T2WI, T2-weighted imaging; FLAIR, fluid-attenuated inversion recovery; SWI, susceptibility-weighted imaging; SNR, signal-to-noise ratio; SDNR, signal difference-to-noise ratio. Bonferroni multiple testing correction for p-value = 0.0166. *p ≤ 0.05.

### Evaluation for Subthalamic Nuclei Border Delineation

Results of the fixed-points scale for delineation between the STN and the substantia nigra, and the scoring for the lateral border of STN were summarized in Table 5. There are “relatively strong” interobserver agreements according to Cramer’s V coefficients. The delineation between STN and substantia nigra was good on both FLAIR and T2WI with a score of 4 or 5 for 81 STNs on FLAIR and 74 STNs on T2WI. But the scoring on SWI was relatively lower with a score of 4 or 5 in less than half STNs (*n* = 36). The Cramer’s V coefficient is 0.406 between FLAIR and T2WI, 0.298 between T2WI and SWI, and 0.193 between FLAIR and SWI. For the lateral border of the STN, the Cramer’s V coefficient is 0.448 between FLAIR and T2WI, 0.221 between T2WI and SWI, and 0.234 between FLAIR and SWI.

**TABLE 5:** Results of the fixed-points scale scores for delineation between the STN and the substantia nigra, and the scoring for the lateral border of the STN.

		The delineation between the STN and the substantia nigra					Delineation of the lateral border of the STN				
Score		1	2	3	4	5	1	2	3	4	5
**FLAIR**											
Right	(*n* = 45)	0	1	5	22	17	0	1	6	21	17
Left	(*n* = 45)	0	1	2	22	20	0	2	7	23	13
Total	(*n* = 90)	0	2	7	44	37	0	3	13	44	30
**T2WI**											
Right	(*n* = 45)	0	2	7	29	7	0	2	14	22	7
Left	(*n* = 45)	1	1	5	30	8	1	5	15	18	6
Total	(*n* = 90)	1	3	12	59	15	1	7	29	40	13
**SWI**											
Right	(*n* = 45)	1	5	20	19	0	3	9	17	13	3
Left	(*n* = 45)	1	6	21	17	0	2	11	16	12	4
Total	(*n* = 90)	2	11	41	36	0	5	20	33	25	7

## Discussion

**FIGURE 6:**
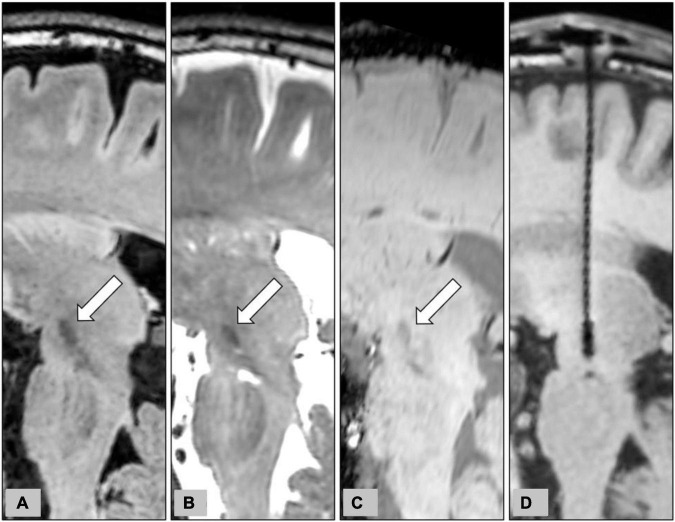
Representative case to show the delineation of STN on different MR pulse sequences for the planning of STN-DBS surgery. The planned trajectory for the right STN-DBS lead was demonstrated on the oblique sagittal reconstruction of pre-operative 3D FLAIR **(A)**, T2WI **(B)**, and SWI **(C)**. The target structures, the STN, were indicated by white arrows on pre-operative images. The final position of the electrode was also shown on the oblique sagittal reconstruction of post-operative T1WI **(D)**.

Indirect targeting of the STN with CT coordinates for stereotactic localization is rapid (Spiegelmann and Friedman, 1991); however, anatomic details of the STN on CT are poor when compared with MRI (Lemaire et al., 1999). On the contrary, although good for visualization of the target structure, 3D reformation, and trajectory planning on conventional 2D MRI is difficult due to the large slice thickness and the non-negligible gap between images. The CT-MR images fusion procedure combines the stereotactic accuracy of CT and the precise anatomical definition of 3D MRI with distortions less than 1 mm except at the tissue-air interface (Kooy et al., 1994). In our institution, we primarily used the direct targeting method with CT-MRI image fusion for STN-DBS surgery. Figure 6 demonstrates the delineation of STN on different MR pulse sequences along the trajectory of DBS electrodes.

In our study, the contrast and the SDNR of the STN were both highest on FLAIR, suggesting the optimal visualization of the STN. FLAIR is one of the inversion recovery sequences used to enhance contrast by selective suppression of water signals. Because the characteristic hypointense SIs of the STN on T2-weighted MR pulse sequences, reflecting the shortened T2 relaxation time by the high intrinsic iron content high in STN, T2WI, and FLAIR are commonly used to target the STN (Dormont et al., 2004). Previous studies reported optimal demonstration of the STN on 3D T2WI at both 1.5 T and 3.0 T (Dormont et al., 2004; Slavin et al., 2006). One previous study compared two-dimensional (2D) T2WI and 3D FLAIR images for the visualization of brain stem anatomy (Kitajima et al., 2012). Another study compared 3D FLAIR with 2D T2-turbo-spin-echo (TSE) and 2D T2*-fast-field echo for the delineation of the STN (Heo et al., 2015), but to our knowledge, no study has yet compared 3D FLAIR with 3D T2WI or 3D SWI for the visualization of the STN in such a large group of patients with medication-refractory PD. It is generally accepted that 3D FLAIR provides high spatial resolution and a high SNR (Li et al., 2020). 3D FLAIR also emphasizes the T2-weighted contrast effect compared with the T2-TSE pulse sequence because of the longer time-to-echo, higher turbo factor number, and longer echo train length (Kitajima et al., 2012). In addition, 3D MR images with whole-brain coverage and thin slice thickness are more suitable for image co-registration than using 2D MR images.

Susceptibility-weighted imaging is a combination of phase and magnitude images with enhanced contrast that is sensitive to hemorrhage, calcium, iron storage, and slow venous blood (Beriault et al., 2014). The high iron concentration of the STN corresponds to increased susceptibility and the hypointense signal on SWI (Dormont et al., 2004). SWI has been reported to be more accurate than T2WI for visualization of the STN at both 1.5 T and 3.0 T field strength (O’Gorman et al., 2011; Kerl et al., 2012; Liu et al., 2013). A previous study reported a higher contrast-to-noise ratio on SWI than that on FLAIR images at 3.0 T (Kerl et al., 2012). However, a 2D axial FLAIR sequence with thick slice thickness (4 mm) was used in that study, and only nine healthy volunteers and one patient with PD were included. Our study also demonstrated a higher SDNR on SWI than that on T2WI. The enhanced visualization of cerebral veins is another benefit of SWI for preoperative planning of DBS lead trajectory. However, the non-local susceptibility effect, also known as blooming artifact, is a notable drawback of SWI. This means that on SWI, the STN may also appear to originate from surrounding non-STN tissue (Li et al., 2012). This blooming artifact, therefore, requires quantification and correction before the accurate direct targeting of the STN.

In our study, the SDNR on SWI was significantly lower among patients who had been on a medication treatment regimen for ≥13 years. The SDNR also trended lower in patients with longer medication treatment on FLAIR and T2WI, but the difference was non-significant. The effect of tissue iron concentration on MR SI is well known, but the exact relationship between iron accumulation and long-term medication treatment in patients with PD remains unclear (Jellinger, 2012). These results in our study suggest a possible change of tissue iron concentration in STN after long-term dopamine-enhancing agent therapy. However, further histopathology study or quantitative measurement by non-invasive imaging is required to verify this postulation.

The delineation between STN and substantia nigra, and the visualization of the lateral border of the STN on different MR pulse sequences were evaluated using a fix-point scale modified from similar research (Suther et al., 2018). The ordinal fixed-point scales, which are composed of ordered quantitative features ranging from “totally disagree” to “totally agree”, are commonly used in subjective image quality assessment for diagnostic images (Bourdel et al., 2015). For the total 90 STNs in 45 patients, scoring for the delineation between STN and substantia nigra was good on both FLAIR and T2WI. The Cramer’s V coefficient suggested a “relatively strong” association between FLAIR and T2WI. But the scoring on SWI was relatively lower, and was only “weakly” associated with FLAIR, and “moderately” associated with T2WI according to Cramer’s V coefficient. These results suggested an inferior ability to delineate between STN and substantia nigra on SWI. For the visualization of the lateral border of the STN, our results also showed a similar disadvantage of SWI.

This study has several limitations. First, we used standardized procedures of ROI placement and SI measurement by two neuroradiologists in this study. However, some errors during ROI placement are still possible. Second, the SNR, contrast, and SDNR are commonly used quantitative parameters for MR image quality and contrast-generating ability. These parameters, however, cannot completely reflect the subjective delineation of STN by the human eyes on MRI. Third, MR image distortion is another challenge for stereotactic surgery and is not covered in this study. Fusion of MRI and stereotactic CT with a metallic frame was performed to overcome the MR image distortion in our hospital. Furthermore, a recent study has suggested that the error of measurement on MRI was random and did not appear to move in any predictable manner (Simon et al., 2005). MR images distortion may not be as significant as it was postulated to be. In addition, this study was based on quantitative analyses of the signal intensity measured on 3D MRI. The STN-DBS surgery is a minimally invasive procedure and relies on stereotactic navigation and multichannel microelectrode recording (MER). Unlike conventional open surgery, the target structures are not directly visualized during operation. In the absence of a golden standard reference, it is difficult to evaluate the clinical accuracy and reliability of STN delineation on different MR pulse sequences.

In conclusion, we reported a retrospective cohort that included preoperative MRI in 45 patients with medication-refractory PD. The contrast and SDNR for the STN are highest on FLAIR, suggesting the optimal ability for delineating STN on MRI. These results can contribute to facilitating the STN delineation during preoperative planning and enhanced electrodes placement accuracy during surgery.

## Data Availability Statement

The datasets presented in this study can be found in online repositories. The names of the repository/repositories and accession number(s) can be found below: https://drive.google.com/file/d/1K8Y_0OkJYrHDWhjzSayBbg10_MVzF8qz/view?usp=sharing.

## Ethics Statement

The studies involving human participants were reviewed and approved by Chang Gung Medical Foundation Institutional Review Board RB No. 202101300B0. Written informed consent for participation was not required for this study in accordance with the national legislation and the institutional requirements.

## Author Contributions

C-YS, AW, and C-HY contributed to the conception and design of the study and performed the data post-processing. C-YS organized the database and wrote the first draft of the manuscript. C-HY performed the statistical analysis. AW, C-CC, P-HT, and CCC wrote sections of the manuscript. All authors contributed to manuscript revision, read, and approved the submitted version.

## Conflict of Interest

The authors declare that the research was conducted in the absence of any commercial or financial relationships that could be construed as a potential conflict of interest.

## Publisher’s Note

All claims expressed in this article are solely those of the authors and do not necessarily represent those of their affiliated organizations, or those of the publisher, the editors and the reviewers. Any product that may be evaluated in this article, or claim that may be made by its manufacturer, is not guaranteed or endorsed by the publisher.
